# Measuring the Quality of Life in Patients with Multiple Sclerosis in Clinical Practice: A Necessary Challenge

**DOI:** 10.1155/2013/524894

**Published:** 2013-02-28

**Authors:** Karine Baumstarck, Laurent Boyer, Mohamed Boucekine, Pierre Michel, Jean Pelletier, Pascal Auquier

**Affiliations:** ^1^EA3279, Self-Perceived Health Assessment Research Unit, School of Medicine, Aix-Marseille University, 27 Boulevard Jean Moulin, 13385 Marseille Cedex 05, France; ^2^Department of Neurology, Timone University Hospital, 27 Boulevard Jean Moulin, 13385 Marseille Cedex 05, France

## Abstract

While the physical disability aspect of multiple sclerosis (MS) is of great importance, quality of life (QoL) measurements are being considered increasingly important with regard to evaluating disease progression, treatment, and the management of care provided to MS patients. Despite the acknowledged need to consider QoL issues, QoL assessment remains underutilized in clinical practice. These issues should be explored and understood to promote the use of measuring QoL in MS clinical practice. We explore the difficulties for clinicians: choosing and determining the most appropriate QoL measure and how to best integrate QoL measurements into clinical practice. This paper discusses several avenues to provide to clinicians arguments of the clinical relevance and accuracy of QoL instruments and ultimately to enhance the use of QoL measures in clinical practice for MS patients.

## 1. Introduction

While the physical disability aspect of multiple sclerosis (MS), the most common demyelinating disease of the central nervous system in young adults, is of great importance, it is now well recognized that it does not reflect all of the facets that patients consider important in their life. Fatigue, depression, and physical disability are only one aspect of a person's experience with MS; it is well documented that cognitive, emotional, and psychological functions contribute to their quality of life (QoL) [1]. The QoL measurements are being considered increasingly important with regard to evaluating disease progression, treatment and the management of care provided to MS patients [2, 3]. The US Food and Drug Administration (FDA) and the European Medicines Agency encourage the use of QoL assessment in patients with chronic illnesses [4, 5], and several groups have published detailed recommendations for QoL assessment [6, 7]. In MS research, 118 studies that have reported QoL as an outcome were performed with MS patients in the Clinical Trials registry (ClinicalTrials.gov, December 31, 2012). Despite the acknowledged need to consider QoL issues, QoL assessment remains under-utilized in MS clinical practice [8]. QoL assessment may be considered to be an “unfulfilled promise” [9–11]. Therefore, these issues should be explored and understood to promote both the use and usefulness of measuring QoL in MS clinical practice. Here, we explore the difficulties for clinicians to choose and determine the most appropriate QoL measure, to be convinced by the clinical utility of the QoL assessment implementation in clinical practice and to interpret QoL scores. 

## 2. Difficulties for MS Clinicians to Choose and Determine the Most Appropriate QoL Measure

QoL is commonly assessed using self-reported questionnaires [3]. To fully understand and explore the effectiveness of any intervention for the management of MS, it is important to have robust, valid, reliable, and universally applied measures [12]. Generic instruments are generally used to compare QoL across different populations, while disease-specific instruments focus on particular health problems and are more sensitive for detecting and quantifying small changes [13]. In MS clinical practice, MS-specific questionnaires are more appropriate due to a better ability to discern QoL differences in patients than the 36-Item Short Form [14].

### 2.1. A Large Variety of QoL Instruments in MS

A large number of disease-specific QoL instruments have been validated for use in MS patients. The most popular questionnaires are the Multiple Sclerosis Quality of Life questionnaire (MSQOL54) [15], the Functional Assessment of Multiple Sclerosis questionnaire (FAMS) [16], the Hamburg Quality of Life Questionnaire in Multiple Sclerosis (HAQUAMS) [17], the Quality of Life Index-Multiple Sclerosis (QLI-MS) [18], the Multiple Sclerosis Quality of Life Index (MSQLI) [19], the Leeds Multiple Sclerosis Quality Of Life scale [20], the MS Impact Scale (MSIS-29) [21], the Disability and Impact Profile (DIP) [22], the Extension of Quality-adjusted Time without Symptoms of Disease and Toxicity of Treatment [23], and more recently, the Multiple Sclerosis International Quality of Life questionnaire [24]. While some reviews tried to describe the different questionnaires as designed specifically for MS patients [2, 25, 26], a clinician contemplating these various rules and instruments may be overwhelmed by their level of complexity. The multiplicity of scales used requires describing their psychometrics and the theoretical and conceptual foundations [27]. Clinicians should be provided better guidance and training that includes evidence of the respective contributions of the various available instruments, the degree to which the tools measure what they claim to measure, and their respective strengths and shortcomings.

### 2.2. The Psychometric Properties of the QoL Measures: Validity, and Reliability, Sensibility to Change

High-level requirements for development and metric validation of QoL measures, especially among the most recent instruments, are now well acknowledged [28, 29]. The definitions of the main psychometric properties are summarized in Box 1. Briefly, we can mention some limitations about the process of validating the QoL questionnaires that may compromise the robustness of the instrument.

First, one important issue concerns the conceptual problems related to the definition of QoL. The researchers should have well-validated questionnaires based on a clear conceptual basis for QoL. One major challenge to explaining the content of the QoL dimensions to be measured is to ensure that the subjects' perceptions are accurately taken into account. Interviews with patients are commonly considered as the best method to capture the patient's perceptions [30, 31] and provide the content of the questionnaire. Few MS-specific QoL questionnaires were exclusively based on the patient's point of view [24].

Second, the “responsiveness” or “sensitivity to change,” defined as the ability to detect a meaningful change, is a core psychometric property of a measuring instrument. Examination of responsiveness requires longitudinal data collection. Given the availability of many QoL instruments, little research has been conducted to test the responsiveness of the QoL tools in MS. The HAQUAMS showed satisfactory responsiveness to change [32], the MSIS-29, MSQOL-54, and FAMS moderately detected change in health status [33, 34]. Also, clinicians should prefer the use of the HAQUAMS to detect health changes over time of MS patients. Future studies should provide comparisons with responsiveness indices using a direct head-to-head comparison to make the situations in which they were tested comparably.

Finally, another point that should be mentioned is related to the number of available languages of the questionnaire. The MSQOL54 [15, 35–37] and the MusiQoL [24, 38–41] are both available in many languages. These questionnaires were developed simultaneously in a number of countries and thus represent a major strength. These 2 instruments can be applied internationally. 

### 2.3. The Acceptability of the Questionnaire

Environmental barriers have been described [42] to explain why QoL measures have not been routinely implemented in clinical practice. Time and resource are both constraints on clinicians whose main role is providing patient care [43]. A great asset of the QoL questionnaire is its acceptability, which concerns the ergonomics of the questionnaire, such as the length of the questionnaire, the paper or electronic format, and the concept of computer adaptive testing.

Some authors have suggested that questionnaires intended for use in clinical populations should be as brief as possible because of the nonadaptability with a clinical evaluation and the difficulties of the concentration and perception faced by patients with a cognitive dysfunction [8, 30], such as MS patients. It is common to accept that the average time of completion of a questionnaire should not exceed 10 minutes to be fully compatible with clinical practice. Providing shorter questionnaires in MS QoL measures, as is already done in other chronic diseases [44], may contribute both appropriate and useful for use in clinical practice.

A potential opportunity for questionnaire development exists in the growing use of electronic records and e-health research [45]. To our knowledge, there are not any studies that evaluate the feasibility of e-form QoL questionnaires in MS patients. However, it is not certain that e-form questionnaires would allow for obtaining QoL data in an efficient real-time manner because of the logistics feasibility and the lack of computer stations and hand-held devices [46].

While most QoL questionnaires are initially fixed in content and length, future challenges now focus on the concept of computer adaptive testing. The number of items can be reduced substantially by use of item-response theory and computer adaptive testing to target questions through an iterative process in which responses determine which items are subsequently presented. This approach requires development and validation of algorithms in addition to development and validation of the original questionnaire [12]. Today, the Neurology Quality-of-Life Measurement Initiative is a standardized approach based on extant items used for measuring QoL across common neurologic conditions, including multiple sclerosis, for both adults and children [47, 48]. This approach allows for comparison of data from different studies.

## 3. Key Arguments for the Clinical Utility of the QoL Measure

The next challenge is to develop credible strategies for integrating QoL data in clinical practice [9]. To enhance the use of QoL measures in clinical decision making, more work is necessary to convince clinicians of the clinical relevance of QoL instruments. Improving knowledge about the determinants of QoL changes and the potential predictive role of QoL on disability may reinforce the conviction of clinicians to use these measures in their MS clinical practice. In the same way, demonstrating that QoL feedback should improve health status of MS patients may confirm the relevance of including QoL in clinical practice. 

### 3.1. Knowledge of QoL Determinants in MS Patients

Clinicians can use QoL assessments to check whether interventions have been as effective from the patient's point of view as from the clinician's, and to determine whether further action is required [2]. Knowledge of which factors are determinants of QoL in patients with MS would assist clinicians in choosing the most appropriate interventions. Several determinants of QoL have been identified with varying strengths of association and include both disease-related variables (disability status [49, 50], disease duration [50, 51], fatigue [52, 53], depression [49, 54]), cognition [50], sociodemographic variables (age and sex [55, 56], level of education, and marital status [50]). A number of these factors might be amenable to treatment intervention, which might be expected to improve QoL: fatigue [57], depression [58], and cognition [59]. 

### 3.2. Knowledge of the Predictive Role of QoL on Health Status

Predictive factors of long-term disability in patients with MS were also previously reported [60, 61]: sociodemographic variables [62, 63], initial EDSS score or initial change in EDSS score [61, 64], number or types of relapses [61, 62], nature of the initial symptoms [65], and MRI findings [66]. The weight of these factors is poorly understood and does not explain the entire change of disability that is observed. In contrast to domains such as heart disease and cancer, few studies have examined the predictive value of QoL on disability in patients with MS. Longitudinal studies have described whether the QoL level, in addition to conventional clinical and sociodemographic factors, provides prognostic information about the evolution of disability in patients with MS [67–70]. These studies have found that scores of mental health QoL [67, 69], scores of “physical-like” dimensions [68, 69, 71], and the score of global QoL [70] are independent predictors of disability as assessed using the EDSS score. There must be at least one plausible mechanism responsible for the link between poor QoL and progression in disability. QoL could be a more subtle measure of early disability that is not detected by the EDSS scale [70].

The identification of early predictors of the long-term evolution of disability status may be useful to identify both high-risk patients who require early and more aggressive therapies and low-risk patients who could avoid lifelong, expensive, and potentially troublesome treatments. Thus, this identification procedure may favor a more homogeneous selection of patients for clinical therapeutic trials [72]. Patient-reported baseline QoL levels provide additional prognostic information on MS disability beyond traditional clinical or sociodemographic factors. These findings provide strong support for the integration of QoL into clinical practice, in addition to other standard assessments, and reinforce the importance of incorporating a patient's evaluation of their own QoL level during patient monitoring and the assessment of treatment effects. Future studies should provide data from longer follow-up times and will likely highlight other robust findings. 

### 3.3. The Impact of QoL Feedback to Clinicians in Clinical Practice

The impact of QoL assessment on health status and other health-related outcomes of patients has already been accomplished in oncology [73–75]. To our knowledge, there are no studies that have explored the effect of assessing QoL in MS care management. The nocebo effect of QoL assessment without feedback should also be considered by clinicians. This effect is defined by the negative expectations that derive from a clinical encounter and lead to poor health outcomes and therapy adherence [76]. This theme constitutes an important avenue of MS research in clinical settings for the coming years. 

## 4. Difficulties in Interpreting QoL Scores

In some specific situations, clinicians can be perplexed when interpreting QoL scores: (1) what does a QoL score mean in the absence of normative/reference values? (2) what does a change in QoL score over time mean? and (3) what is the meaning of QoL scores for an individual with cognitive impairment?

### 4.1. The Lack of Norms in MS QoL Scores

The practical and clinical interpretations of QoL data in a given disorder are difficult unless these data are presented with a reference system. One of the difficulties encountered when interpreting a QoL score for clinicians is the lack of norms values. SF36, a generic instrument, is commonly used because normative data from healthy adults and individuals with a variety of illnesses are available [77]. To our knowledge, no norms were provided for any MS-specific questionnaire. At this time, the QoL scores of the reference population described in the validation publication are implicitly used as norms. It is rare to have scores according to sex, gender, and clinical form. Additionally, it becomes imperative to produce norms for the most popular MS-specific instruments. Aggregating datasets may contribute to produce valid and robust norms. Each patient would be compared to norms.

### 4.2. The QoL Changes over Time: The Question of Response Shift

Another concern expressed by clinicians is the interpretation of QoL measures in longitudinal studies because QoL, self-reported by the patient, might be influenced by psychological phenomena such as adaptation to illness. Adaptation to illness is a potential explanation in cases where, for example, the QoL of an individual who has experienced a serious health event or chronic condition is similar to the QoL of a healthy individual. Most people with a long-term chronic condition such as MS do not say that physical disability is their primary concern but mention involvement in everyday activities and psychological and emotional well-being [1]. An important mediator of this adaptation process is “response shift” (RS) which involves changing internal standards, values, and the conceptualization of QoL [78, 79]. These changes do not allow comparing QoL changes over time. RS can be divided into (1) reconceptualization (i.e., a redefinition of QoL), (2) reprioritization (i.e., a change in the importance attributed to component domains constituting QoL), and (3) recalibration (i.e., a change in a patient's internal standards of measurements). True change may be over- or underestimated when RS is present, leading to biased estimates of the magnitude of change. A recent meta-analysis revealed a substantial body of literature on RS phenomena and concluded that RS was common and significant in QoL measurement [80]. Some studies have already investigated this phenomenon in MS populations using the most established methods [78]: the then-test, structural equation modeling (SEM) [81], latent trajectory analysis of residuals [82], recursive partitioning tree analysis as a data mining method [83], and, more recently, the random forest method [84]. Each method has its own specific advantages and limitations that have been clearly discussed [85]. It would be premature to conclude which method is best for detecting RS in MS patients. The variety of methods developed illustrates the complexity and difficulty in detecting RS. Future explorations should be performed to compare the capacity of these methods for detecting RS and the degree of convergence of the isolated phenomena. However, the RS does not necessarily invalidate QoL measures when it appears under the reprioritization component. Change in values may simply represent a mechanism by which people gain true changes in QoL [86]. Determining how to integrate the RS in the interpretation of QoL scores in MS clinical practice is now the next challenge.

### 4.3. QoL Scores among MS Populations with Cognitive Dysfunction

Prior studies of the relationship between cognitive impairment and QoL have been contradictory, highlighting either negligible [87–90] or strong links [51, 91, 92] between cognitive disturbances and QoL alterations. The use of self-reported outcomes in subjects with cognitive dysfunction is of particular concern [93]. The extent to which MS patients with cognitive dysfunction can validly self-report their QoL is a crucial issue that has only partially been examined. While some authors argue that cognitively impaired individuals are unable to produce valid QoL measures [94, 95], others reported empirical evidence suggesting that individuals with a moderate degree of cognitive impairment can perform reliable QoL assessments [92, 96]. Two recent papers reported data providing strong arguments to support the conclusion that MS patients with executive dysfunction, as determined by the Stroop test [97], and memory dysfunction, as determined by the Grober and Buschke test [98], are reliable and consistent when answering a well-validated MS-specific QoL questionnaire, the MusiQoL [24, 38]. These studies provided new evidence about the suitability for using self-reported QoL data in these specific populations. The assessment of QoL using the MusiQoL questionnaire could be more widely used without concern over the adequacy of this approach for cognitively impaired patients. However, it has to be acknowledged that a single test of cognitive functioning will never be entirely appropriate. An interdisciplinary approach would be most effective in addressing this deficit [1, 12]. Future studies should provide similar results according to other definitions of cognitive dysfunction that integrate combinations of different composites (i.e., memory, attention, and concentration) and other QoL questionnaires.

## 5. Conclusion

Using QoL measures may provide clinicians with information regarding the general health status of their MS patients who might otherwise go unrecognized. Neurologists should consider QoL measures in the same way as routine objective measures such as symptomatic evaluation scales, laboratory tests, and radiographs to manage the care of MS patients [46]. In this paper, we discussed several avenues to convince clinicians of the clinical relevance and accuracy of QoL instruments and ultimately to enhance the use of QoL measures in clinical practice for MS patients.

## Figures and Tables

**Box 1:**
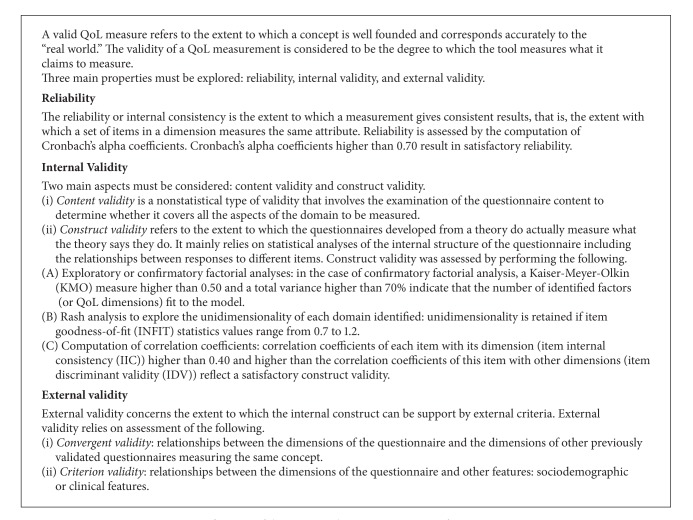
Definitions of the main psychometric properties of a QoL measure.
